# Effects of Increased Spatial Complexity on Behavioural Development and Task Performance in Lister Hooded Rats

**DOI:** 10.1371/journal.pone.0047640

**Published:** 2012-10-16

**Authors:** Sophie J. Lyst, Katherine Davis, John Gigg, Reinmar Hager

**Affiliations:** Faculty of Life Sciences, University of Manchester, Manchester, United Kingdom; Université Pierre et Marie Curie, France

## Abstract

Enhancing laboratory animal welfare, particularly in rodents, has been achieved through environmental enrichment in caging systems. Traditional enrichment such as adding objects has shown to impact development, reproductive and maternal performance as well as cognition. However, effects of increased spatial complexity as part of larger novel caging systems have not been investigated. While adoption of caging systems with increased spatial complexity seems uncontroversial from a welfare perspective, effects of such housing on the development and task performance of experimental animals remains unclear. In this study, we investigate differences in key behaviours and cognitive performance between Lister Hooded rats housed in traditional (single-shelf) cages (‘basic’) and those housed in larger cages with an additional shelf (‘enriched’). We found minor differences in maternal behaviour, such as nursing and offspring development. Further, we compared task performance in females, using a hippocampus-dependent task (T-maze) and a hippocampus-independent task (Novel Object Recognition, NOR). While in the T-maze no differences in either the rate of learning or probe trial performance were found, in the NOR task females housed in enriched cages performed better than those housed in basic cages. Our results show that increased spatial complexity does not significantly affect development and maternal performance but may enhance learning in females for a non-spatial task. Increased spatial complexity does not appear to have the same effects on behaviour and development as traditional enrichment. Thus, our results suggest no effect of housing conditions on the development of most behaviours in experimental animals housed in spatially enriched caging systems.

## Introduction

Most behavioural research is conducted in lab-housed animals due to the significant advantages of a controlled environment. Such laboratory animal facilities are typically designed to provide a standardised environment where animals can be kept in good physical health, whilst at the same time considering economic costs. Rodents used in research are typically housed in small cages that lack key features of their natural environment. These conditions have been shown to impose constraints on brain development, particularly in the hippocampus and restrict naturally occurring behaviour, which leads to altered brain function [1]. This, in turn, may have implications for the validity and conclusions drawn from experimental data when using rodents in research, especially in behavioural neuroscience [2]. Recognising the limitations of artificial housing has led to many attempts to improve these conditions through ‘environmental enrichment’ [3]. In order to qualify as enrichment, Leach et al. [4] suggested that any change to the housing system should increase the frequency and diversity of positive natural behaviours, decrease the occurrence of abnormal behaviours, maximize the utilization of the environment and increase the animal's ability to cope with the challenges of captivity.

Commonly, environmental enrichment involves adding novel objects to cages, along with nesting material or shelters (see Simpson and Kelly [5] for review) but it can also be considered in terms of the cage design. The Code of Practice for the Housing and Care of Animals in Designated Breeding and Supplying Establishments [6] discusses aspects of housing for rodents and mentions environmental improvement in terms of arranging the cage volume to create additional complexity through extra floor space (e.g., tubes) and platforms. Earlier studies by Chamove [7] showed that increasing the complexity of the environment within a cage can be beneficial to animals. These studies in mice showed that individuals raised in more complex environments were more active and more inclined to explore novel situations. Similar studies assessing levels of environmental enrichment have shown that when given the choice, rodents will spend proportionately more time in the more complex environment than the barren one, suggesting a preference for enrichment [2], [7], [8], [9].

In an effort to enhance the welfare benefits of caging systems for laboratory rodents, a novel cage type for rats has been developed that provides more floor space and an additional raised platform, thus, increasing spatial complexity. This allows rats to display natural behavioural traits, such as a bi-pedal posture. The platform also provides a shelter on the ground level, which has been shown previously to promote welfare in rodent housing [10]. However, the effect of this change in environmental complexity (over traditional housing) on behavioural development and maternal performance remains unclear. Additionally, while the benefits of the new, large cage type to general animal welfare seem apparent, there has been considerable debate about the impact of this environmental change on performance in experimental paradigms and comparability with data collected from animals kept in traditional caging systems [11]. To date, no study has attempted to quantify possible changes in development and performance in key tasks associated with housing in spatially enriched cages.

General environmental enrichment and increased spatial complexity has been shown to impact on hippocampal-dependent memory [12], [13], improve spatial awareness [14] and increase concentrations of key neurotrophic factors [15]. Frequently, the effects of environmental enrichment on cognition have been studied through spatial memory assessment in the water maze [16], [17]. While this paradigm allows for direct testing of hippocampal-dependent processes, it is also susceptible to confounds, for example, anxious animals adopting a thigmotaxic search strategy [17]. For this reason, we applied another hippocampal-dependent task, the T-maze, to examine appetitive learning for the spatial location of an edible reward [18]. If there is a positive impact on hippocampal development from being housed in a larger, spatially complex (‘enriched’) cage, we predict that these individuals should show increased spatial awareness in the T-maze than those animals housed in the smaller, traditional (‘basic’) style of cage. For comparison, the Novel Object Recognition task (NOR) was applied to examine the effect of cage type on object memory, a non-spatial process frequently considered to be hippocampus-independent [19], [20].

In this study, we first raised and housed rats in either enriched or basic cages and then investigated the effect of cage type on their development and maternal performance. Secondly, we tested the hypothesis that enrichment in the form of increased spatial complexity has a developmental effect on the brain, specifically the hippocampus. We predicted that animals in enriched cages would demonstrate superior performance than those animals in basic cages in the T-maze since this is a hippocampus-dependent task. By contrast, we predicted no difference in performance in the Novel Object Recognition task (hippocampus-independent).

## Methods

### Ethics Statement

Principles of laboratory animal care in this study were in accordance to UK Home Office Guidance regulations, with approval from the University of Manchester ethical committee board under permit number 40/3409.

### Subjects

Six female (180–200 g) and two male (300–320 g) 9-week old Lister-Hooded rats were purchased from Charles River UK to provide the F0 generation. A pigmented strain of rat was chosen due to the visually-guided nature of the cognitive tasks and an expectation that this strain has a high level of visual acuity. These F0 animals were then randomly assigned to experimental conditions by cage type (basic or enriched) and used to breed the F1 generation, which were the subjects of the experiment. We mated a total of 18 F1 females (9 for each of the two cage types) and 6 males (3 each), which were selected using a random number function computer application. The F1 females were then bred with non-litter males and, once pregnant, housed individually. Upon birth, we recorded maternal behaviour and offspring development until weaning of the F2 at 21 days of age (d21). After d21, the F1 females were housed in groups of three during the cognitive testing, whilst the males remained individually housed to prevent aggression. Maternal and cognitive behaviours in the F1 were used to investigate effects of cage type. All animals were housed in a temperature-controlled environment on a 12-h light/dark cycle with the lights on from 07:00 h–19:00 h. All animals were given *ad lib* access to food pellets and water throughout the experiment.

### Experimental condition: Cage type basic or enriched

**Figure 1:**
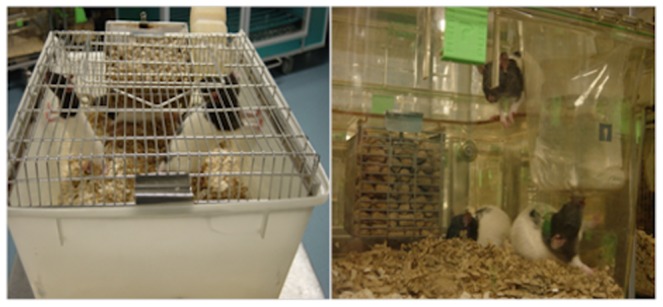
Rats housed in a basic cage (left panel) or in an enriched cage (right panel).

Two cage types, a standard NKP RC2 (‘basic’) and a spatially enriched, double decker, Tecniplast GR1800 (‘enriched’) cage define our two conditions. The basic cage (505 mm×315 mm×185 mm; base) consisted of an opaque plastic base (and a lid of metal bars with an opening at one end for access). Food is placed in a metal trough at one end of the cage and obtained through the metal bars. Water is stored in plastic bottles with a metal nozzle which protrudes through the bars. The cage has only one level and wood chips are used as the base layer, with paper bedding placed in a corner (Figure 1 left panel). By contrast, the enriched cage (380 mm×305 mm×240 mm; base) was made entirely of clear plastic with a shelf that adds an extra dimension to the living space. Food was placed within a metal cage attached to the underside of the shelf and obtained through metal bars. Water was provided in sterile plastic pouches with disposable plastic mouth pieces which fit into specially designed access points in the corners of the cages. As in the basic cages, a base layer of wood chips was used with paper bedding placed in a corner of the cage on the ground level. Each cage was a self-contained unit with individual air supply through connection valves in the back of the cage that connected to fixtures in the metal racks in which they were stored. Temperature and humidity within the cages on each rack was controlled and monitored directly through this system (Figure 1 right panel).

### Developmental and behavioural data

The mating pairs (F0) were housed in their respective cage type as outlined above until females were visibly pregnant at which point they were separated into individual cages. Food intake and body weight was recorded weekly throughout the experiment. At birth (day 1) of the resulting litters (F1), the mother's weight, litter weight and size and amount of food was recorded. On day 6 and 14 these measurements were taken again (in addition to the weekly weights) along with behavioural recordings (see below). Pups (F1) were weaned at 21 days of age and housed by sex until they reached nine weeks of age. A power analysis (GPower3.1.3; *d* = 1.5, α = 0.05, power = 0.80; effect size calculation based on estimated effect sizes in NOR tasks and T-maze) [21], [22] was used to determine that nine individuals per cage type were required for adequate statistical power. For each cage type, a randomly selected set of nine females and three males from the F1 litter were divided into three cages per experimental group in the same 3∶1 non litter-mate harem ratio used with the F0 generation for mating. Then, visibly pregnant (F1) females were separated and housed individually, as were the males, in their respective cage type. We recorded maternal behaviour of the F1 females nursing their F2 offspring and body weight data were then recorded at days 2, 6, 10 and 14 until eighteen days of age. Thus, we were able to compare maternal behaviour between females who themselves were raised in either basic or enriched cages. We used the F1 males and females further for T-maze and Novel Object Recognition testing (below).

### Maternal behaviour

Behavioural observations were carried out when the pups were 6, 10, 14 and 18 days of age. To standardise the conditions which the mothers were exposed to prior to collection of behavioural data, a separation technique was used, based on an established protocol developed for the study of provisioning in mice [23], [24]. This ensures that mothers are motivated to exhibit maternal behaviour and offspring are motivated to suckle, independent of variation due to differences in observation time [25].

Mothers were separated from their litters for four hours, after which they rejoined their pups and maternal behaviour was recorded for 15 minutes. First, the weight of the mother, litter, weight of food and litter size were recorded (we refer to this time point as ‘-4 hours’). The mother was then placed in a separate cage of the same type as her experimental condition (i.e. females in basic cages were placed in a separate basic cage) with the food and water from the original cage. The pups were left in the original cage which was placed on a heat mat. After 4 hours, weight measurements were taken again and the mother placed back in the cage with the pups (time = ‘0 hours’). The mother and young were then observed for 15 minutes using focal sampling [26] with a record of behaviour taken every 20 secs. A specifically developed ethogram, which included a full repertoire of behaviours observed in a pilot study under such conditions, was used to record behaviour. After these 15 minutes, the mothers were left with their young undisturbed for 1 h45 mins. Two hours after mothers rejoined their pups, body weight and food intake were again recorded (time =  ‘+2 hours’) and the food in the cage was topped back up to 500 g.

### T-maze task

#### Apparatus

**Figure 2:**
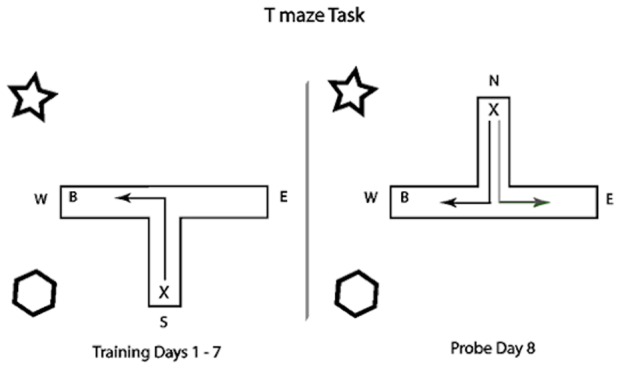
T-maze apparatus with distal cues (star and hexagon symbols) located in the north west and south west. The ‘X’ indicates the starting position of the rat and ‘B’ indicates the location of the food reward. In the left panel for training days 1–7, the black arrow indicates the fastest path required to reach the reward (a left turn). In the right panel, the grey arrow indicates a learned stimulus response (body turn) whereas the black arrow shows the place response (a right turn).

The maze was made of black 5 mm thick Perspex (Gilbert Curry UK) with three 30 cm×10 cm arms. On the floor of each arm, green, non-reflective plastic was applied to prevent reflected light from startling the animals. The south arm was used as the start arm for all training trials. At the end of the east and west arms was a glass container 10 cm in height with a black plastic bottle cap on top to act as a food hopper. The maze was located in a behavioural testing room and surrounded by black cloth screens on all sides to obscure the investigator and control spatial cues. In the two corners to the left of the starting arm (north west and south west) children's soft toys were pinned to the screen to act as distal visual cues to the rats (Figure 2).

#### Habituation to T-maze

Prior to starting the T-maze protocol, rats were habituated to the apparatus to prevent neophobia of a novel environment. On 3 consecutive days, rats were placed into the T-maze in the south arm and allowed to explore the maze for 5 minutes. Food treats were placed in the bottle caps in both arms of the maze to encourage feeding and exploration within the maze. After each day of habituation, rats were given 2 food treats to consume in their home cages to increase their preference for the reward. T-maze training began only after habituation had finished and when rats showed no visual signs of distress of being in the maze. Rats were not food restricted prior to training or testing.

#### T-maze protocol

Rats were tested using a T-maze protocol adapted from Packard and McGaugh [18] with 7 days of training and a probe trial on day 8. On each day during training, the food reward was placed only in the goal arm (west arm) of the T-maze, which was located closest to the distal cues. Rats were given four food rewarded trials per day for the seven day training period. In each trial, rats were released into the south arm of the T-maze and were allowed up to 2 mins to traverse the maze and consume the food treat in the goal arm. If their first entry into an arm was the west (baited) goal arm, a correct response was recorded. First entries into the east (un-baited) arm were marked as errors during training. To assess learning over the seven testing days, a score of 0.25 was given to each trial which had a correct first arm entry, up to a maximum value of 1 (4 trials×0.25), per day per rat. Incorrect first arm entries were given a score of zero. A correction procedure was used so that rats, which made an incorrect response, were then allowed to enter the baited arm and consume the food treat. In cases where the animal failed to consume the food treat within 2 mins, the trial was terminated and the animal placed in the baited arm and allowed to consume the food. After the completion of each trial, the animal was placed back in their home cage which was located directly behind the starting arm of the maze and allowed a 1 min interval before the next trial.

To determine whether rats were using either a spatial or a stimulus-response based strategy to navigate the T-maze, a single probe trial was carried out for each animal on day 8. On this probe trial, the T-maze was rotated by 180 degrees so that the start arm was now facing north. Care was taken to ensure that the spatial positions of the arm ends were identical to those for the training trials. The reward was relocated from the previously baited west arm (which now faced east) to the newly positioned west arm, now closest to the distal cues. The animals were placed in the north facing start arm of the maze and allowed to make a single entry into either the baited or the un-baited maze arm. Those animals using a ‘place’ hippocampal strategy to navigate the maze were expected to turn right, towards the distal cues to gain the reward. Animals using only a stimulus-response strategy (i.e. turn left for the reward in the learning phase), were expected to turn left, as in training to seek the reward in the arm now facing east. Correct probe trials were awarded a value of one whereas an incorrect arm entry received a value of zero. The number of correct versus incorrect probe responses on day 8 were compared between individuals born and housed in basic and enriched cages. We predicted that animals raised in a spatially enriched environment would perform better in this task than those animals in basic cages, based on the assumption that enrichment affects hippocampal development.

### Novel Object Recognition task (NOR)

#### Apparatus

The task was carried out in an area constructed from 5 mm white Perspex (Gilbert Curry, floor dimensions 100 cm×100 cm, with 25 cm walls). The arena was surrounded by external spatial cues. Stimuli were wooden blocks (5 cm×5 cm×5 cm) of different colours fixed together using superglue into shapes and were adhered to the floor of the arena using blue-tack^R^. Identical copies of each object were constructed in triplicate and these different copies were used in each acquisition and test phase to control for olfactory cues. A new set of three copies plus one extra novel object were used for each NOR trial repeat. All phases of testing were videotaped using a Logitech webcam, and object exploration was timed offline using stopwatches.

#### Habituation

To investigate the performance of animals in a non-hippocampal dependent task, we used the Novel Object Recognition task with time delays of one or four hours in a protocol adapted from Ennaceur and Delacour [27]. Prior to starting the NOR task, individuals were habituated to the arena over five days. On days 1 and 2, animals were placed in cage mate groups (females) or individually (males) in the arena and were allowed to explore for 5 mins. To prevent object neophobia, two objects were placed centrally in the area allowing the animals to see and explore stimuli. On days 3–5, all animals were returned to the arena individually for ten minutes. All objects used during habituation stages were then discarded. Between different rats, objects were cleaned with 70% ethanol solution to remove all odour cues; at the end of the daily testing the arena was thoroughly cleaned. Animals were run in the same order each day to keep odour cues in the arena constant for the next animal. The arena was also cleaned with 70% ethanol solution between animals. The same cleaning protocol was applied to NOR testing.

#### NOR Protocol

**Figure 3:**
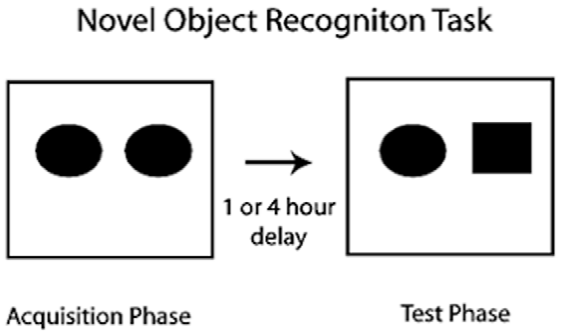
Novel Object Recognition task protocol. Animals experience a 3 mins acquisition phase, a variable ITI day and a 3 mins test phase. At test, the novel object (square object) on the right replaces the familiar object from the acquisition phase.

Each task comprised a 3 mins acquisition phase, followed by an ITI (inter-trial-interval) delay of one or four hours, and a further 3 mins test phase. Each rat was tested at each time delay twice, such that it experienced the NOR for four times (2×ITI and 2×repeat) in total. The ITIs of one hour and four hours were chosen to represent both a short and a long term delay for the NOR. In the acquisition phase, rats were placed in the area and allowed to explore for 3 mins. Within the arena, two identical objects were placed: one on the left and one on the right to the centre. Rats were then removed from the arena, and returned to their home cage for the delay of one or four hours. At test, rats were returned to the arena and allowed to explore for a further 3 mins. The two acquisition phase objects were replaced by a copy of the original object and a novel object (Figure 3).

The left/right position of novelty at test was balanced such that each rat experienced it on the left once, and the right once for each of the two ITIs. Testing took place for one trial per day over four consecutive days, beginning with one hour ITI on days 1 and 2, and four hours delay on days 3 and 4. The time (in seconds) each rat spent exploring objects on the left and right positions for both acquisition and test phases were recorded from video recordings offline using a stopwatch [27]. ‘Exploring’ was defined as time spent actively sniffing or investigating the object whilst in direct contact. For the acquisition phase, the total time spent exploring the left and right objects were added to provide a measure of exploration and to ensure all rats had satisfactorily explored both objects. We found no significant difference of total exploration time between animals housed in the two conditions (GLM, F_1,22_ = 0.97, p = 0.34). Both sets of animals had explored the left and right objects for a satisfactory period. From this, we were able to show that animals in the basic cage type were not neophobic and NOR could be carried out reliably. A displacement index (D2) was calculated for the test phase following D2 = (N−F)/(N+F), where N = time (s) exploring the novel object and F = time (s) exploring the familiar object. A value of +1 demonstrates sole exploration of the novel object whereas a value of 0 indicates no object preference. Increased differential exploration of the novel object was taken as an indication of intact object recognition memory [22], [27]. Total object observation time at test (the time in seconds spent exploring both left and right objects) was also analysed as a measure of activity.

### Data analysis

We analysed developmental and behavioural data using linear models and t-tests where appropriate, using SPSS version 16.0. T-maze performance in the F1 was analysed using a 4 by 7 Mixed ANOVA to compare the performance depending on cage type (basic or enriched) versus day of training (days 1–7; used to assess incremental learning), and to account for differences due to sex.

To analyse the NOR data, a 2 by 2 Mixed ANOVA with Bonferroni post-hoc tests was conducted to examine the effect of cage type (basic or enriched) versus ITI delay (1 or 4 hours). Further, the displacement index D2 of each group was compared to a chance value of 0 using one sample t-tests to confirm that each group was able to identify the novel object. We further investigated sex dependency and, again, male and female D2 values were compared to chance (‘0’) to confirm performance. To identify whether there were any differences in object exploration during the test phase the total time of observation of both objects was analyzed in a further 2 (cage type) by 2 (ITI delay) Mixed ANOVA.

## Results

### Development and growth

**Figure 4:**
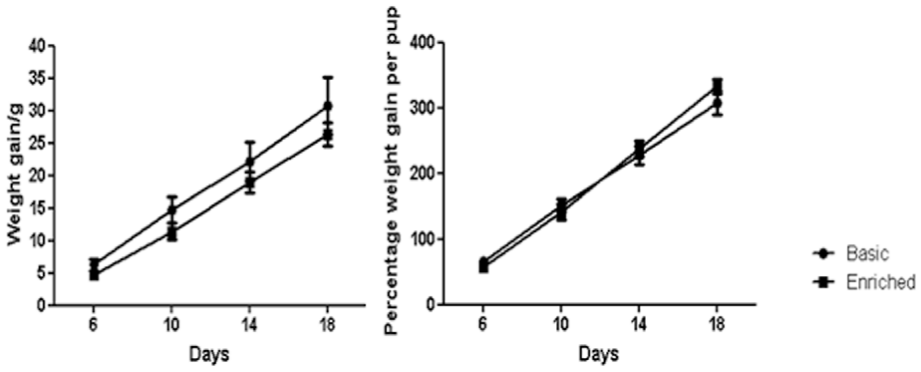
Average relative growth rates of individual pups in basic and enriched cages (left panel) and shown as percentage weight change (right panel).

We calculated average growth per pup from day 2 to day 18. Figure 4 (left panel) shows absolute growth rates as weight gain in grams. While average growth rates in the basic cage were slightly higher than those in enriched cages, these differences were not statistically significant (GLM, F_1,36_ = 0.12, p>0.05). When adjusting for initial differences in body weight, however, no differences can be distinguished between the groups (Figure 4 right panel).

### Food intake and litter size

**Figure 5:**
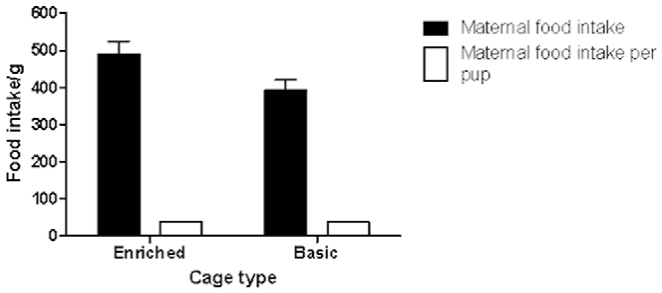
Maternal food intake from birth of litter until 14 days of age.

We compared maternal food intake during the first 14 days of lactation (after which pups may start eating solid food) between females in basic and enriched cages. There was a significant difference, with those in the enriched cages consuming significantly more food in the 14 days after the birth of the litter compared to those in basic cages (GLM, F_1,36_ = 9.770, p<0.01; Figure 5). However, after accounting for differences in litter size, differences in intake were non- significant as indicated by the per pup food consumption. The average litter size in enriched cages was 13.44 while it was 11.22 in basic cages, a non-significant difference (t_16_ = 0.11, p>0.05).

### Behavioural data

**Figure 6:**
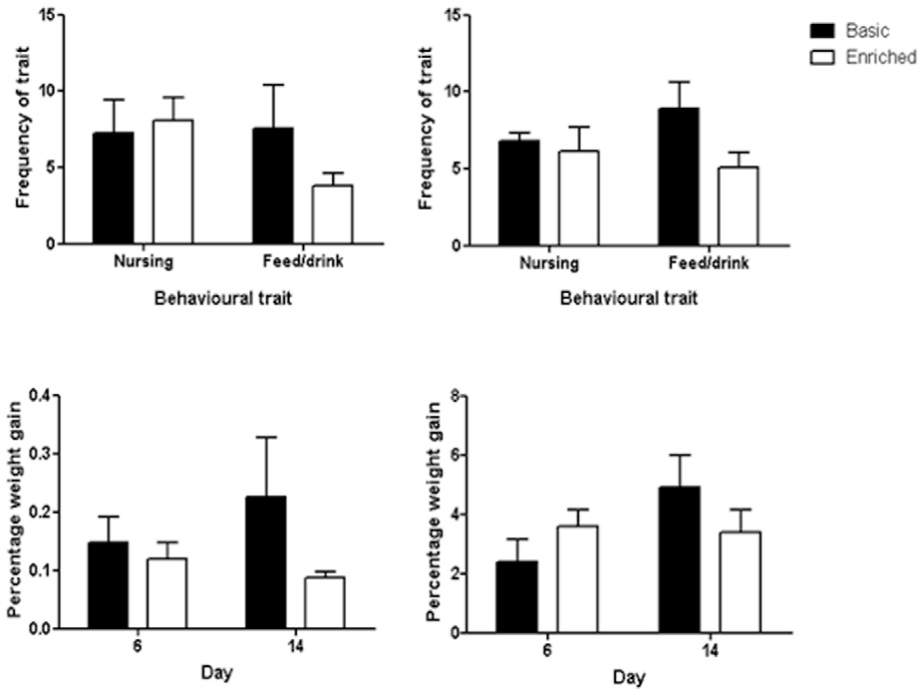
Frequency of nursing and feeding/drinking traits of females in the two cage types on day 6 (top left panel) and day 14 (top right panel) of lactation. Weight gain of litter during a 2 h period after rejoining their mother on day 6 (bottom left panel) and day 14 (bottom right panel).

We focused on nursing (i.e. mothers seen suckling) as the key maternal behaviour and differences in feeding/drinking (non-maternal behaviour) between females in the two cage types on two days during lactation (day 6 and day 14). There was a significant difference in feeding/drinking, but not nursing, between females. Figure 6 (top left and right panels) show that females housed in the basic cages spent significantly more time feeding and drinking (GLM, F_1,36_ = 4.43, p = 0.043) than those in enriched cages

We further analysed the weight gain of the litter for a two hour period after the mothers rejoined their litter on day 6 and day 14 (during which all mothers were motivated to show maternal behaviour). When correcting for initial pup weight differences on the respective day and using the mother's weight as a covariate, no significant difference was found for the average percentage weight gain of all pups between the two cage conditions (F_1,36_ = 3.58, p = 0.068) although differences are nearing significance with pups nursed by mothers in enriched cages show a higher weight gain (Figure 6 bottom left and right panels).

### T-Maze results

We analyzed the results of the T-maze task in two stages: First, the rate of learning was assessed by comparing the proportion of correct trials per day for each rat (n = 12 in each condition, males and females) in the respective cage types over the seven days of training. Second, we compared performance in the probe trial (correct scored ‘1’, incorrect scored ‘0’) to assess whether rats were able to demonstrate the use of a spatial ‘place’ strategy upon facing a new start point.

**Figure 7:**
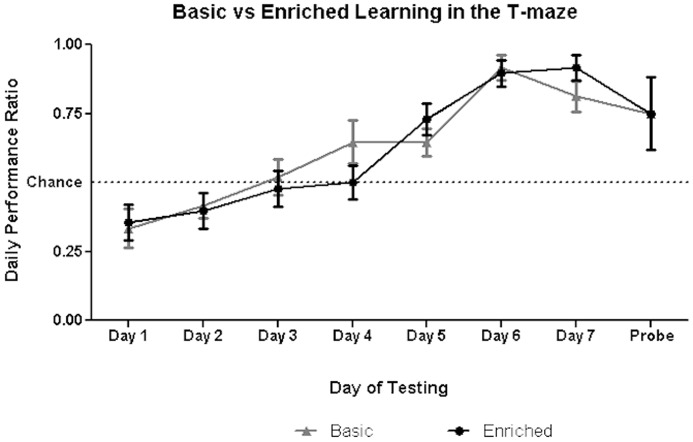
T-maze performance on days 1–7 of training and day 8 (probe). Chance performance of 0.5 is depicted by the dotted line.

T-maze learning was analyzed using a 2 (cage type) by 7 (days of training) Mixed ANOVA. While there was a significant effect of training day (F_7,160_ = 12.99, p<0.001), there was no effect of cage type (F_3,160_ = 0.14, p>0.05) nor an interaction effect between cage type and day (Figure 7). There was further no effect of sex on learning. Thus, both individuals housed in basic and enriched cages learn to navigate in a hippocampal-dependent task at the same rate.

Probe trial data on day 8 were analyzed in a 2-tailed unpaired t-test. There was no significant difference in the performance between the two groups (t_22_ = 0.13, p>0.05). Analyzing males and females separately we found, again, no significant differences between cage types.

### Novel Object Recognition Results

**Figure 8:**
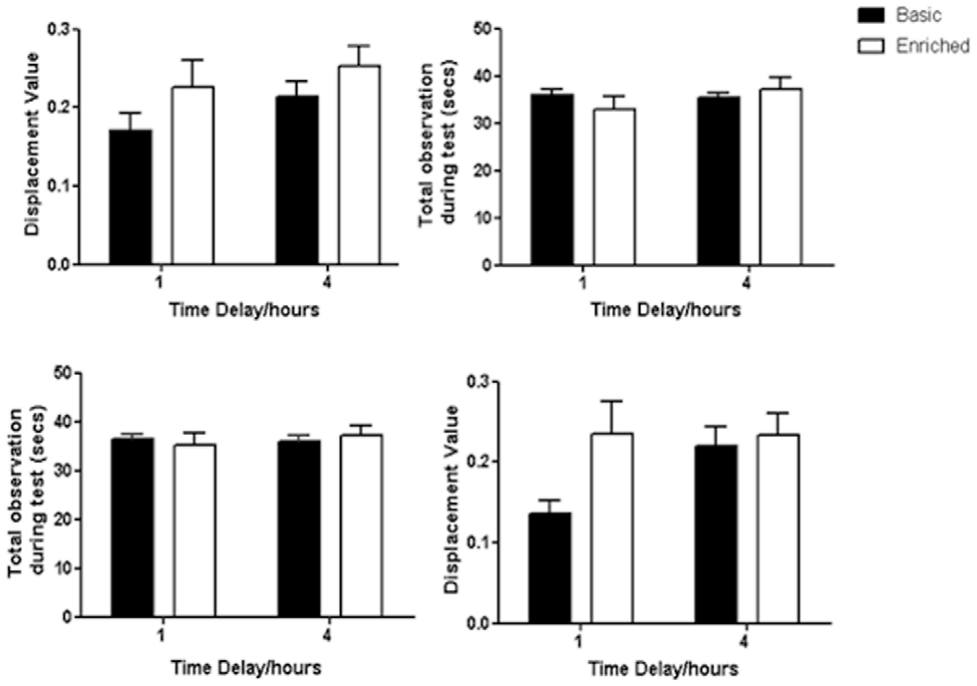
Novel Object Recognition task. Displacement index (D2) values for the two cage types and time delays (top left panel) and level of object observation between the delays and cage conditions (top right panel) The same information is shown for female data only in the bottom right and bottom left panels respectively.

We analyzed NOR data using a two (cage type) by two (ITI) Mixed ANOVA. There were no significant differences in performance (Figure 8 top left panel) or total observation (Figure 8 top right panel) for rats in basic versus enriched cages (n = 24). Thus, there was no effect of cage type (F_1,22_ = 2.97, p>0.05), ITI (F_1,22_ = 1.92, p>0.05) nor an interaction between cage type and ITI when comparing D2 values (F_1,22_ = 0.01, p>0.05; Figure 8 top left panel). Further, there was no significant difference between cage types when analysing the total duration of object observation (amount of time spent exploring both objects; ‘total observation’) between cage types (Figure 8 top right panel), nor during the acquisition phase. The D2 performance of each cage group was compared to chance (‘0’) for each ITI using paired t-tests. All groups were able to perform the task at a significantly higher level than expected by chance (t_11_ = 6.57 and higher, p<0.001 in all cases).

Looking at sex-specific effects, there was no significant difference in D2 values between males from the two cage types, a cage type difference approached significance for females (F_1,16_ = 3.73, p = 0.071). There were no significant differences for either ITI (F_1,16_ = 2.18, p>0.05) or an interaction (F_1,16_ = 2.35, p>0.05), however there was a near significant difference between females from enriched and those from basic cages at the 1 h interval (F_1,16_ = 3.73, p = 0.071; Figure 8 bottom right panel). There were no significant differences in female total observation at either ITI (Figure 8 bottom left panel), suggesting that the better performance of enriched females at ITI 1 h was not simply due to increased object observation. In conclusion, while both basic and enriched cage types could perform above chance in this novel object recognition task, it was only through separating out the sexes that an effect of cage was seen.

## Discussion

Whilst most examples of environmental enrichment focus on the addition of novel objects and nesting material to caging, the effect of general housing dimensions and enrichment in terms of cage design has not been investigated to date. For the first time, we have directly examined the behavioural effects of housing animals in a spatially enriched cage with two levels when compared to animals housed in basic cages. Overall, our results show very few differences in development, maternal performance and behaviour between animals raised and housed in basic and enriched cages. If increased spatial complexity has the same effects as traditional enrichment on hippocampal development, we predicted a difference in performance in a hippocampus-dependent task and no differences in a hippocampus independent task. Our results clearly show that enriched spatial complexity does not have the same effect as traditional enrichment on the hippocampus as there was no difference between rats in basic and enriched cages in T-maze task performance. Further, we did not detect any difference between cage types in their effect on hippocampus-independent task performance in the NOR; all animals could perform the task at both delays although females (but not males) from enriched cages seemed to perform marginally better in this task. Overall, our results suggest that spatially enriched cages may be substituted for the traditional basic cage type, without affecting the comparison to research conducted on animals housed in a basic cage type.

### Behaviour

Focusing on behavioural differences between mothers housed in either basic or enriched cages, we detected only an effect of cage type in feeding and drinking behaviour. We used a comprehensive and established ethogram [24] covering all key behaviours (maternal and non-maternal), thus, it is unlikely we missed any behaviours for which females in the two cage types might differ. Females in basic cages spent significantly more time performing feeding and drinking than females in enriched cages in a situation where they had just rejoined their litters and were expected to show nursing behaviour. Interestingly, there is no difference in maternal behaviour, which suggests that increased feeding and drinking does not go at the expense of performing maternal behaviours (and thus the fitness of the pups). Rather, females that spent more time drinking were less active otherwise and females from both enriched and basic cages showed similar levels of maternal behaviour.

Any differences in food consumption can be explained by the larger average litter size in enriched cages (13.44 (enriched) vs 11.22 (basic)), which, although not significant with our sample size, may suggest that breeding in enriched cages may be more productive. This may be due to the enriched cages being individually ventilated so that no pheromones from any other animal in the room (especially males) can be detected. Being fully enclosed there are also fewer disturbances to pregnant females from other activities in the lab including cleaning.

### Task Performance

Previous research has suggested that raising animals in a traditionally enriched environment promotes physiological and neurochemical changes in the hippocampus and increases spatial awareness [13], [14], [15]. Assuming that spatial enrichment has the same effect as traditional enrichment, we predicted differences in task performance between rats in the two cage types in a hippocampus-dependent task such as the T-maze, but no difference in a hippocampus-independent task such as Novel Object Recognition (NOR). In contrast to our predictions, we found no differences in spatial memory between the two cage groups in the T-Maze. Previous research has indicated that animals in basic cages show more errors and require a larger number of trials in maze tasks to reach a learning criterion than animals from enriched cages [28], [29], however, in our animals; we found no indication of ‘basic’ animals being disadvantaged. Further, in our study most rats from either cage type were able to find the food reward upon rotation of the maze by 180 degrees. Therefore it appears that animals from both cage types had been navigating via distal cues to traverse the maze. Harris et al. [17] found animals housed in basic cages to use a thigmotaxic search strategy in water maze, perhaps as a manifestation of anxiety to a novel environment [7]. When removing the thigmotaxic animals from the data, they found no differences in the performance of basic or enriched groups. Overall, our results of no difference in T-maze performance suggest that enrichment in terms of increased spatial complexity may be different from traditional enrichment in their effects on task performance.

Turning to the NOR task, performance in which is generally considered independent of the hippocampus [19], [20], we found that animals from both cage types explored the objects equally at test and there were no differences in the performance of both groups at recognising the novel object. However, when analysing by sex there was a significant difference in the effect of cage type on task performance. Females, but not males, from enriched cages performed significantly better than those raised in basic cages at the 1 hour time delay. It has recently been argued that there is a time-dependent involvement of the hippocampus in NOR memory [30], [31]. Specifically, delays longer than 3 hours are impaired by damage to the hippocampus whereas shorter delays remain unimpaired without a functioning hippocampus. In our study, we found improved performance in enriched females at the shorter delay, in contrast to our prediction of a positive effect on hippocampal development in enriched animals. It is generally considered that the perirhinal cortex, adjacent to hippocampus is critical in mediating object recognition memory (see Winters et al. [32] for a comprehensive review). Therefore, it is possible that enriched females have a developmental change in the perirhinal cortex that confers an advantage at short time intervals. Future studies are required to establish whether this underlies the differences found between animals from basic and those from enriched cages at the short time delay in the NOR task. Our results emphasize the importance of analyzing acquisition data from NOR experiment in advance to assess if differences in such traits exist between conditions and sexes.

## Conclusions

In summary, the enriched cages were not shown to significantly improve spatial awareness or affect object recognition memory when considering both sexes together. This deviates from findings of previous research examining traditional enrichment effects and we thus conclude that enhanced spatial complexity as implemented in double-decker caging systems does not have equivalent effects on task performance as traditional enrichment.
